# Post-traumatic stress disorder and associated factors among inpatients at Eastern Command Referral Hospital in Dire Dawa, Eastern Ethiopia

**DOI:** 10.3389/fpsyt.2024.1373602

**Published:** 2024-06-11

**Authors:** Desalegn Adugna, Tesfaye Assebe Yadeta, Jerman Dereje, Dawit Firdisa, Samuel Demissie Darcho, Obsan Kassa, Monas Kitessa, Asefa Tola Gemeda

**Affiliations:** ^1^ School of Public Health, College of Health and Medical Science, Haramaya University, Harar, Ethiopia; ^2^ Department of Nursing, School of Nursing and Midwifery, College of Health and Medical Science, Haramaya University, Harar, Ethiopia; ^3^ Department of Psychiatry, College of Health and Medical Science, Haramaya University, Harar, Ethiopia; ^4^ School of Pharmacy, College of Health and Medical Science, Haramaya University, Harar, Ethiopia

**Keywords:** post-traumatic stress disorder, traumatic events, armed forces of military members, Dire Dawa, Ethiopia

## Abstract

**Background:**

Post-traumatic stress disorder (PTSD) is characterized by heightened stress and anxiety after experiencing a traumatic event. While numerous studies have been conducted to investigate the magnitude and factors associated with PTSD, there is limited evidence available on specific study populations of military personnel.

**Objective:**

The study aimed to determine the magnitude of post-traumatic stress disorder and associated factors among military personnel admitted to the Eastern Command Referral Hospital in Eastern Ethiopia from May 1 to 30, 2023.

**Methods and materials:**

A cross-sectional study was carried out at an institution. Face-to-face interviews were conducted to collect data using the post-traumatic stress disorder military version checklist for the Diagnostic and Statistical Manual, Fifth Edition. Data were entered and analyzed using EpiData version 3.1 and STATA version 14. Descriptive statistics were employed to summarize the information. To investigate factors linked with outcome variables, bivariate and multivariate logistic regression analyses were conducted. The results were presented using odds ratios with 95% confidence intervals, with statistical significance given at a p-value of 0.05.

**Results:**

This study found that approximately 23.6% (95% CI = 19.9–27.8) of admitted military members fulfilled the diagnostic criteria for PTSD. Participants’ history of mental illness [adjusted odds ratio (AOR) = 5.73, 95% CI = 2.66–12.31], family history of mental illness (AOR = 10.38, 95% CI = 5.36–20.10), current chewing of khat (AOR = 2.21, 95% CI = 1.13–4.32), physical trauma (AOR = 2.03, 95% CI = 1.00–4.13), moderate social support (AOR = 0.27, 95% CI = 0.1–4.53), strong social support (AOR = 0.09, 95% CI = 0.02–0.35), and severe depression (AOR = 2.06, 95% CI = 1.74–5.71) were factors significantly associated with post-traumatic stress disorder.

**Conclusions:**

The magnitude of post-traumatic stress disorder is high among military personnel. Factors such as participants’ history of mental illness, family history of mental illness, depression, lack of social support, current use of khat, and physical trauma are significantly associated with PTSD. It is crucial to identify and intervene early in individuals with these risk factors to address PTSD effectively.

## Introduction

Post-traumatic stress disorder (PTSD) is a common mental health problem for military members (1). This illness can have a substantial influence on all parts of life and is frequently chronic. Public safety workers, such as firefighters and military personnel, are constantly subjected to stressful situations because of the nature of their work (2). They may risk their lives to preserve others or property, resulting in potentially painful events (3). Military personnel deployed in conflict zones endure extra stressors, such as exposure to explosives, which can contribute to the development of PTSD (4). PTSD can develop from experiencing or witnessing life-threatening situations, manifesting as symptoms like flashbacks, nightmares, hyperarousal, avoidance behaviors, and mood changes (5). PTSD frequently coexists with other mental health problems, such as anxiety and depression, among military members (4). The Diagnostic and Statistical Manual of Mental Disorders, Fifth Edition (DSM-5) describes a traumatic event as having encountered a major injury, approaching death, or sexual violence (6).

PTSD is an extensively researched mental health disorder, especially after traumatic events and disasters (7). The likelihood of experiencing PTSD can vary based on factors such as occupation, level of exposure to trauma, and other variables (8). Workers, especially those in the military, are at a high risk for developing PTSD (2). Untreated PTSD can lead to long-term health problems, reduced daily functioning, and an overall decline in productivity, health, and social interactions, placing a burden on society (9–11). A significant portion of disability cases in both developed and developing countries are linked to PTSD (12). While preventing PTSD is difficult, secondary preventative strategies are successful. Treatment can also be helpful for certain individuals, especially veterans (13).

The World Health Organization (WHO) suggests that the magnitude of mental disorders is higher in war settings. PTSD is one of the most commonly reported mental health concerns among veterans and service personnel, accounting for 22.1% of all cases (14). There are 316 million adult war survivors worldwide suffering from PTSD and/or depression, mostly in low- and middle-income countries, with a combined burden of 3 million disability-adjusted life years due to PTSD (15). The occurrence of PTSD, affecting 5% to 20% of the 2.7 million Americans deployed to Iraq and Afghanistan since 2001, is influenced by the degree of battle exposure (16, 17). A study of 613 US soldiers hospitalized after combat injury found that 4.2% had probable PTSD at 1 month, increasing to 12.2% at 4 months and remaining at 12.0% at 7 months (18). Another study of 1,777 US military personnel post-deployment health assessment showed that approximately 25.15% had PTSD (19).

According to Virgil Hawkins’s stealth conflict map, the African continent accounts for approximately 88% of global morbidity and mortality related to conflicts (20). More than 75% of African countries have been engaged in wars in the last three decades, with more than 70% of victims being fighters (21). This has led to significant human losses, infrastructure destruction, and immense suffering for millions of Africans (15, 22). Studies on the magnitude of PTSD in Sub-Saharan Africa have shown estimates ranging from 0% to 74% at national and regional levels, with a combined magnitude of 30% in war-affected areas (23). In Nigeria, recent research indicates that three out of 10 military combatants are at risk of developing PTSD (24).

PTSD is not well understood, especially in relation to military personnel. Studies have shown that individuals can develop PTSD after experiencing traumatic events like landslides and traffic accidents (25). This study intends to fill this gap by assessing the post-traumatic disorder and associated factors among military personnel at the Eastern Command Referral Hospital in Dire Dawa Administration, Eastern Ethiopia, from May 1 to 30, 2023. This study is critical for better understanding and meeting the mental health requirements of military personnel in this region.

## Methods and materials

### Study setting, design, and period

A cross-sectional study was conducted at Eastern Command Referral Hospital in Dire Dawa Administration from May 1 to 30, 2023. Dire Dawa Administration is located 515 km from Addis Ababa in the eastern part of Ethiopia. The hospital serves as the base hospital for casualties and provides family planning, psychiatric, medical, and surgical services for inpatients and medical and surgical services for outpatients. It also offers services to military personnel requiring inpatient or mental care. The hospital has a total of 1,200 beds and a staff that includes 10 medical doctors, 8 emerging surgeons, 12 midwives, 50 clinical nurses, 33 health officers, 9 laboratory technicians, 8 pharmacists, 6 psychiatrists, 1 psychologist, and 25 health extensions (Eastern Command Referral Hospital Report, 2023).

#### Source population, study population, and eligibility criteria

The source population for this study included all members of the armed forces who visited the inpatient department of the Eastern Command Referral Hospital in the Dire Dawa Administration. The study population consisted of military personnel who had been exposed to battlefields or sustained injuries on the battlefield and sought treatment at the hospital during the study period. The study included military members who were admitted to the inpatient department and were able to participate in the questionnaire. Individuals who were unable to communicate or were critically ill were excluded from the study.

### Sample size determination and sampling procedure

This study has two separate objectives: the first is to assess post-traumatic stress disorder, and the second is to identify factors associated with post-traumatic stress disorder. The sample size for the first objective was determined using a single population proportion formula by taking the magnitude of PTSD to be 22% from a study conducted in Northwest Ethiopia, Bahir Dar (26). With a 4% margin of error, the 95% confidence interval was calculated as n = Z^2^p (l − p)/d^2^, where d is the margin of error, Z is the confidence level (95%) = 1.96, and p is the population proportion = 0.22.


$$\text{n}=\frac{{\left(1.96\right)}^{2}\text{X}\left(0.22\times 0.78\right)}{{\left(0.04\right)}^{2}}=412$$

Accounting for a 10% non-response rate, the final sample size was adjusted to 453. For specific objective 2 focusing on the factors associated with PTSD, the sample size was determined using EPI-Info version 3.1 software. The calculations were based on assumptions of 80% power, 95% confidence interval, and a 1:1 ratio of cases to controls sourced from a previous study (26). The sample size for the first objective was larger than that of the second, resulting in a final sample size of 453.

Participants in the study were chosen using a simple random sampling technique. A sampling frame was created by naming all military personnel from each ward and assigning bed numbers. Four data collectors designated the bed numbers 2 days prior to data collection. Then, study participants were selected using a computer-generated random number.

### Data collection procedure and quality control

Data were collected by three BSc nurses and one BSc psychiatry professional using a structured questionnaire derived from previous studies (27–31). The questionnaire was initially prepared in English, translated into Amharic, and then back-translated to English to ensure clarity. It included sociodemographic, psycho-social, and clinical factors. Face-to-face interviews were conducted with participants at a convenient time in the hospital, with on-site supervision by the principal investigator. Any doubts or errors in the collected data were resolved immediately. The completeness of the data was also checked.

Prior to the actual data collection study, a pretest was conducted outside the study area with 5% of the calculated sample size. Data collectors underwent a 2-day training session. Strict supervision and cross-checking of data were implemented for quality control. The supervisor and principal investigator conducted daily inspections to ensure completeness and quality of data collection, providing detailed feedback to data collectors. Feedback from data collectors was used to enhance the questionnaire. The principal investigator reviewed all questionnaires collected each day, addressing any unclear, missing, or confusing information with the respective data collector for correction.

### Operational definitions

#### Post-traumatic stress disorder

The dependent variable was measured using 17 items from the PCL-M for DSM-5. This self-report rating scale assesses PTSD symptoms based on the DSM-5 criteria. Participants rate each item on a 5-point Likert scale (0 = not at all, 1 = a little bit, 2 = moderately, 3 = quite a bit, and 4 = extremely). The total score was calculated by summing the ratings of the 17 items, resulting in scores ranging from 0 to 85. A cutoff point of ≥50 was used to indicate the presence of PTSD symptoms (32).

#### Social support

The level of social support was assessed using the Oslo-3 social support scale, which assigns scores ranging from 3 to 14. Poor social support was indicated by scores of 3 to 8, moderate social support by scores of 9 to 11, and strong social support by scores of 12 to 14 (31).

#### Depression

The participants were assessed using the Patient Health Questionnaire-9 (PHQ-9) questionnaire, which consists of nine items. Scores were categorized as follows: minimal (1–4), mild (5–9), moderate (10–14), severe (15–19), and very severe depression (20–27) (33).

#### Anxiety disorder

Anxiety levels were assessed using the Generalized Anxiety Disorder 7-item (GAD-7) scale. The cutoff points of 5, 10, and 15 were used to classify anxiety levels as none/normal (0–4), mild (5–9), moderate (10–14), and severe (15–21). Participants scoring<10 on the GAD-7 scale were considered to have anxiety, while those scoring 8 or higher were deemed to have significant anxiety symptoms (34).

#### Family history of mental illness

To assess family history of mental illness, respondents were asked if they had any family members who had been clinically diagnosed with a mental illness (34).

### Data processing and analysis

The data collected were coded, entered, and cleaned using EpiData Software version 3.1. Subsequently, the data were exported and analyzed using STATA version 14. Descriptive statistics such as means, frequencies, percentages, and standard deviations were computed with STATA version 14 and presented in the form of numbers, texts, and tables. Initially, each variable underwent bivariate logistic regression analysis to identify associations with the dependent variable. Variables with a p-value of<0.25 were then included in multivariate logistic regression for further analysis. Variables with a p-value<0.05 in the multivariate models were considered significantly associated with the dependent variable and determined using adjusted odds ratios with 95% confidence intervals. The model’s goodness of fit was assessed using the Hosmer–Lemeshow test.

## Results

### Sociodemographic characteristics of participants

A total of 445 military personnel were included in the study out of the 453 sampled participants, resulting in a response rate of 98.2%. The average age of the participants was 24.8 years with a standard deviation of 6.31. The majority of the respondents (87.4%) were male, and most of them (76.4%) were single. More than half (55.5%) identified as orthodox, and the majority (69.2%) had a secondary educational status. A significant portion (61.1%) held a soldier/army military rank, and the majority (89.7%) belonged to the branch of the military force of the earth mechanized (**Table 1**).

**Table 1:** Sociodemographic and military characteristics among military members at Eastern Command Referral Hospital, Eastern Ethiopia, 2023 (n = 445).

Characteristics		Frequency (N)	Percentage (%)
Age	18–24	281	63.1
	25–29	67	15.1
	30 and above	97	21.8
Sex	Male	389	87.4
	Female	56	12.6
Religion	Orthodox	247	55.5
	Muslim	118	26.5
	Protestant	72	16.2
	Others	8	1.8
Marital status	Single	340	76.4
	Married	89	20
	Divorced/widowed	16	3.6
Educational status	Cannot read and write	25	5.6
	Primary school	112	25.2
	Secondary and above	308	69.2
Average monthly income (ETB)	1,651–5,250	128	28.8
	5,251–7,800	190	42.7
	Over 7,801	127	28.5
Year in service	≤10	397	89.3
	>10	48	10.7
Military rank	Soldier/army	272	61.1
	Non-commissioned officer (NCO)	112	25.2
	Line officer	49	11
	Senior officer	12	2.7

### Clinical, substance use, and psycho-social factors of participants

Approximately two-fifths (44.3%) of the participants had ever used alcohol, and approximately one-third (29.7%) had ever used khat. Also, 26.3% of participants had a family history of mental illness, while 59.8% had a history of chronic medical illness. Among those with a chronic medical condition, 30.1% had a history of mental illness. Additionally, 61.4% of participants screened positive for depression. Moreover, approximately 14.6% of the participants reported having strong social support, while approximately 41.4% of the respondents indicated that they have three to five close individuals who they can rely on for help (**Table 2**).

**Table 2:** Clinical, substance use, and psycho-social factors of the military members at Eastern Command Referral Hospital, Eastern Ethiopia, 2023 (n = 445).

Variables	Category	Frequency (N)	Percentage (%)
Had been diagnosed with chronic medical condition	Yes	266	59.8
	No	179	40.2
Family history of mental illness	Yes	117	26.3
	No	328	73.7
History of mental illness	Yes	92	20.7
	No	353	79.3
Depression	Minimal	172	38.6
	Mild	177	39.8
	Moderate	59	13.3
	Severe	37	8.3
Anxiety	Normal	150	33.7
	Mild	191	42.9
	Moderate	91	20.5
	Severe	13	2.9
Social support	Poor	121	27.2
	Medium	259	58.2
	Strong	65	14.6
Substance use	Alcohol	197	44.3
	Khat	132	29.7
	Smoking	112	25.2

### Exposure to traumatic event factors and magnitude of PTSD

**Figure 1:**
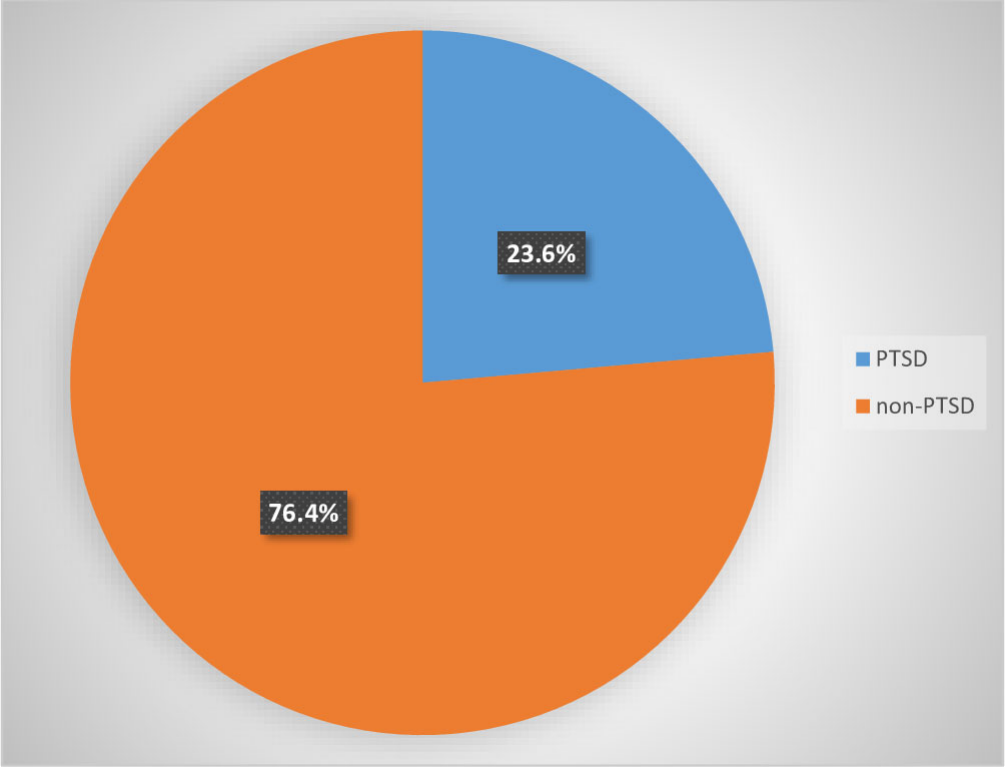
Magnitude of PTSD among military members at Eastern Command Referral Hospital, Eastern Ethiopia, 2023 (n = 445). PTSD, post-traumatic stress disorder.

The majority of respondents (96.4%) stated that they were without food or drink, and almost 94.4% claimed to have witnessed a friend or family member being killed. Approximately 67.9% of the respondents had physical trauma or serious injury, and approximately 61.4% experienced destruction of personal property (**Table 3**). Approximately one-quarter of the participants, 23.6% (95% CI = 19.9–27.8), had PTSD (**Figure 1**).

**Table 3:** Exposure to traumatic event factors of the military personnel, Eastern Command Referral Hospital, Ethiopia, 2023 (n = 445).

Exposure to traumatic events	Category	Frequency (N)	Percentage (%)
Destruction of personal property	Yes	273	61.4
	No	172	38.7
Lack of food or water	Yes	429	96.4
	No	16	3.6
Murderer of family member	Yes	396	88.9
	No	49	11.1
Witnessing murder of family member/friend	Yes	420	94.4
	No	25	7.6
Ill health without medical care	Yes	432	97.1
	No	16	2.9
Tortured or beaten	Yes	61	13.7
	No	384	86.3
Heavy noise of weapons/explosion	Yes	75	16.8
	No	370	83.2
Physical trauma/serious injury	Yes	302	67.9
	No	143	32.1
Imprisonment against your will	Yes	92	20.7
	No	353	79.3

### Post-traumatic stress disorder by clinical, substance use, and psycho-social factors

Among respondents, patients with known chronic medical illnesses, compared to those without, have a higher proportion of PTSD (67.6% vs. 42.6%, respectively). Respondents who have been exposed to heavy noise of weapons, compared to those who have not been exposed, have a higher proportion of PTSD (32% vs. 21.9%, respectively). Also, respondents who have a history of chewing khat, compared to those without, have a higher proportion of PTSD (29.3% vs. 21.4%, respectively; **Table 4**).

**Table 4:** Post-traumatic stress disorder by clinical, substance use, and psycho-social factors among the military personnel, Eastern Command Referral Hospital, Ethiopia, 2023 (n = 445).

Variables	Category	PTSD	
		Yes (%)	No (%)
Religion	Orthodox	59 (23.9)	188 (76.1)
	Muslim	24 (20.3)	94 (79.7)
	Protestant	20 (26.7)	55 (73.3)
	Others*	2 (25)	6 (75)
Marital status	Single	87 (25.6)	253 (74.4)
	Married	16 (18)	73 (82)
	Divorced	2 (12.5)	14 (87.5)
Diagnosed with mental illness	Yes	38 (36.2)	67 (63.8)
	No	286 (84.1)	54 (15.9)
Family history of mental illness	Yes	66 (62.9)	39 (37.1)
	No	289 (85)	51 (15)
Any known chronic medical illness	Yes	71 (67.6)	34 (32.4)
	No	145 (42.6)	195 (57.4)
Current khat chewing	Yes	36 (29.3)	87 (70.7)
	No	69 (21.4)	253 (78.6)
Educational status	Cannot read and write	3 (12)	22 (88)
	Primary school	22 (19.6)	90 (80.4)
	Secondary school	78 (26.9)	211 (73.1)
	Diploma and above	2 (10.5)	17 (89.5)
Heavy noise of weapons	Yes	24 (32)	51 (68)
	No	81 (21.9)	289 (78.1)
Exposure to physical trauma	Yes	78 (25.8)	224 (74.2)
	No	27 (18.9)	116 (81.1)
Social support	Poor	64 (40.3)	95 (59.7)
	Moderate	38 (28.4)	196 (71.6)
	Strong	3 (5.8)	49 (94.2)
Depression	Minimal	45 (26.2)	127 (73.8)
	Mild	30 (16.9)	147 (83.1)
	Moderate	11 (18.6)	48 (81.4)
	Severe	19 (51.4)	18 (48.6)

PTSD, post-traumatic stress disorder.

Others*: Wakefata or Jehovah.

### Factors associated with PTSD

In bivariate regression, various factors such as sex, age, military rank, service years, marital status, education level, branch of military, salary, medical and mental health history, social support, substance use, traumatic experiences, and exposure to violence were examined for their relationship with PTSD. In multivariate binary logistic regression, after adjusting for other variables, a history of mental illness, family history of mental illness, current use of khat, physical trauma, social support, and depression were found to be significant predictors of PTSD (**Table 5**).

**Table 5:** Bivariate and multivariate analyses of factors associated with PTSD among military personnel in Eastern Command Referral Hospital, Ethiopia, 2023 (n = 445).

Variable	PTSD		COR (95% CI)	AOR (95% CI)
	Yes	No		
Military rank				
Soldier/army	70	202	1	1
Non-commissioned officer (NCO)	26	86	0.87 (0.52–1.46)	0.67 (0.32–1.39)
Line officer	8	41	0.56 (0.25–1.26)	0.54 (0.19–1.57)
senior officer	1	11	0.26 (0.03–2.07)	0.49 (0.04–6.42)
Religion				
Orthodox	59	188	1	1
Muslim	24	94	0.81 (0.48–1.39)	0.53 (09.23–1.11)
Protestant	20	55	1.23 (0.68–2.22)	1.42 (0.63–3.19)
Others	2	6	1.06 (0.21–5.40)	0.28 (0.032–2.58)
Marital status				
Single	87	253	1	1
Married	16	73	0.64 (0.35–1.15)	0.65 (0.28–1.49)
Divorced	2	14	0.42 (0.09–1.86)	0.15 (0.02–1.13)
Ward				
Medical	207	68	1	1
Surgical	128	34	0.81 (0.51–1.29)	0.97 (0.49–1.92)
Psychiatric	5	3	1.83 (0.43–7.84)	2.68 (0.30–23.64)
Diagnosed with mental illness				
Yes	38	67	3.00 (1.83–4.92)	5.73 (2.66–12.31) *
No	286	54	1	1
Family history of mental illness				
Yes	66	39	9.59 (5.84–15.74)	10.38 (5.36–20.10) *
No	289	51	1	1
Any known chronic medical illness				
Yes	71	34	1.55 (0.98–2.46)	1.52 (0.78–5.98)
No	145	195	1	1
Current khat chewing				
Yes	36	87	1.52 (0.95–2.43)	2.21 (1.13–4.32) *
No	69	253	1	1
Educational status				
Cannot read and write	3	22	1	1
Primary school	22	90	1.79 (0.49–6.53)	2.23 (0.46–10.88)
Secondary school	78	211	2.71 (0.79–9.31)	4.05 (0.90–18.26)
Diploma and above	2	17	0.86 (0.13–5.76)	1.23 (0.13–12.19)
Heavy noise of weapons				
Yes	24	51	1.68 (0.97–2.89)	1.77 (0.85–3.70)
No	81	289	1	1
Exposure to physical trauma				
Yes	78	224	1.50 (0.91–2.45)	2.03 (1.00–4.13) *
No	27	116	1	1
Social support				
Poor	64	95	1	1
Moderate	38	196	0.29 (0.18–0.46)	0.27 (0.14–0.53) *
Strong	3	49	0.09 (0.03–0.30)	0.09 (0.02–0.35) *
Depression				
Minimal	45	127	1	1
Mild	30	147	0.58 (0.34–0.97)	0.33 (0.16–1.67)
Moderate	11	48	0.65 (0.31–1.35)	0.29 (0.11–1.79)
Severe	19	18	2.98 (1.44–6.17)	2.06 (1.74–5.71) *

COR, crude odds ratio; AOR, adjusted odds ratio; PTSD, post-traumatic stress disorder.

*Significantly associated, 1: reference group.

The likelihood of developing PTSD was significantly higher among respondents who had a previous diagnosis of mental illness [adjusted odds ratio (AOR) = 5.73, 95% CI = 2.66–12.31]. Similarly, individuals with a family history of mental illness had significantly higher odds of developing PTSD (AOR = 10.38, 95% CI = 5.36–20.10). Current khat users were also more likely to develop PTSD (AOR = 2.21, 95% CI = 1.13–4.32) compared to non-users. Additionally, individuals who had experienced physical trauma or serious injuries had higher odds of PTSD (AOR = 2.03, 95% CI = 1.00–4.13) compared to those who had not been exposed. Participants with strong social support had 91% lower odds of developing PTSD compared to those with poor social support (AOR = 0.09, 95% CI = 0.02–0.35). Similarly, participants with moderate social support had 73% lower odds of developing PTSD (AOR = 0.27, 95% CI = 0.14–0.53) compared to those with poor social support. Additionally, respondents with severe depression had 2.06 times higher odds of developing PTSD compared to those without depression (AOR = 2.06, 95% CI = 1.74–5.71) (**Table 5**).

## Discussion

The study aimed to assess the magnitude of PTSD and its related factors among military personnel hospitalized at the Eastern Command Referral Hospital in Dire Dawa Administration. The research identified several key risk factors for PTSD, including a personal or family history of mental illness, current khat use, physical trauma, level of social support, and depression.

This study found that the magnitude of PTSD among military personnel hospitalized after significant combat injury with complex polytrauma was 23.6% (95% CI = 19.9–27.8). This rate is similar to studies conducted in Nepal (21.9%) (35), Nigeria (25.1%) (36), and the USA (22%) (37). However, it is lower than the rates reported in earlier research on military veterans in South Africa (33%) (38), Uyghur and Han military veterans in China’s Xinjiang region (29%) (39), soldiers with amputation of a limb or spinal injury in Sri Lanka 41.7% (40), New Zealand military personnel (30%) (41), US military service members (47%) (42), and US War Veterans receiving post-deployment VA Health Care (37.8%) (43). Possible reasons for the high magnitude in the study conducted in Sri Lanka, Western countries, the USA (42, 43), and New Zealand personnel (41) may be military personnel who were amputated, have spinal injuries, and have higher rates of anxiety and depression (44), which was associated with PTSD. The possible reason for the lower magnitude of PTSD in the study area compared to previous studies may be that traditional societies, such as Ethiopia, have more frequent intimate family structures and a stronger extended family system than societies in Western countries. According to the study, people who have strong support networks (family and community) are more resilient, are able to cope, and can also minimize some of the psychological impacts of serious battlefield injury events, including PTSD (45, 46).

In this study, individuals with a history of mental illness were found to have 5.73 times higher odds of experiencing PTSD compared to those without a previous diagnosis (AOR = 5.73, 95% CI = 2.66–12.31). This finding aligns with previous research (47) and suggests that pre-existing mental health conditions may exacerbate the development of PTSD. Additionally, the study indicated that individuals with a family history of mental illness had 10.38 times higher odds of developing PTSD compared to those without such a history, consistent with earlier studies (27, 48). This association may be attributed to the increased vulnerability to mental health issues in individuals with a familial predisposition, potentially leading to the onset of PTSD (49).

The study found that individuals who currently chewed khat were 2.21 times more likely to develop PTSD compared to those who did not use khat. This finding is consistent with previous studies conducted in Southwestern Uganda (50), Ethiopia (51), China (52), and Somalia (53). Substance abuse, like khat use, can hinder the body’s natural ability to cope with trauma, increase physiological arousal, and worsen PTSD symptoms (54). Additionally, khat is a stimulant that can heighten anxiety levels, potentially exacerbating PTSD symptoms or increasing susceptibility to the disorder (55).

The study revealed that individuals who had experienced physical trauma or serious injuries were 2.03 times more likely to develop PTSD compared to those who had not been exposed. This finding is consistent with previous research that showed higher rates of PTSD among injured soldiers compared to uninjured soldiers (56). Additionally, individuals hospitalized after physical trauma were found to have elevated rates of both PTSD and major depression (57). Moreover, individuals with a history of prior traumatic events and PTSD were at a higher risk of developing PTSD after subsequent trauma (58). This association may be attributed to the strong connection between peritraumatic dissociation during an injury event and the development of PTSD (59). Furthermore, individuals with coexisting depression are more likely to encounter traumatic situations and are at an increased risk of developing PTSD (60).

The study found that individuals with strong social support had a 91% lower chance of developing PTSD compared to those with poor social support. Similarly, participants with moderate social support had a 73% lower risk of PTSD. These results are consistent with previous research (61–64), suggesting that social support plays a crucial role in mitigating the impact of trauma. Individuals with strong social networks are better equipped to cope with emergencies, while those lacking support may struggle to recover, increasing their vulnerability to PTSD (65). The study found that individuals with severe depression were 2.06 times more likely to have PTSD compared to those without depression. This result is consistent with previous research (42, 66) and may be attributed to the shared symptoms and heightened emotional distress experienced in both conditions (67).

### Limitation of the study

The study’s cross-sectional design may not establish temporal relationships between PTSD and its predictors. The tool was not validated in the local language due to budget constraints. However, steps were taken to ensure data validity, including translating the questionnaire, pretesting it, and training the supervisor and data collector.

## Conclusions

The magnitude of post-traumatic stress disorder is high among military personnel. Factors such as a history of mental illness, family history of mental illness, depression, lack of social support, current use of khat, and physical trauma are significantly associated with PTSD. It is crucial to identify and intervene early in individuals with these risk factors to address PTSD effectively.

## Data availability statement

The raw data supporting the conclusions of this article will be made available by the authors, without undue reservation.

## Ethics statement

Ethical clearance was obtained from the Institutional Health Research Review Committee (IHRERC) of Haramaya University. Formal letters of permission were obtained from the School of Public Health and submitted to the hospital’s responsible authorities. Voluntary, informed, written, and signed consent was obtained from the hospital head. The objectives, significance, benefits, and risks of the study and the procedural details of the study were explained to the study participants. Voluntary, informed, written, and signed consent was obtained from each study participant before conducting the interview. Participants were informed of the purpose of the study, and no identification or names were recorded to maintain confidentiality. Study participants were informed of their right to refuse or stop participating at the time during the interview.

## Author contributions

DF: Conceptualization, Formal Analysis, Investigation, Methodology, Software, Supervision, Writing – original draft, Writing – review & editing. DA: Conceptualization, Data curation, Formal Analysis, Investigation, Methodology, Writing – original draft, Writing – review & editing. TY: Supervision, Writing – original draft, Writing – review & editing. JD: Formal Analysis, Investigation, Methodology, Writing – original draft, Writing – review & editing. SD: Formal Analysis, Investigation, Methodology, Writing – original draft, Writing – review & editing. OK: Formal Analysis, Investigation, Methodology, Writing – original draft, Writing – review & editing. MK: Formal Analysis, Investigation, Methodology, Writing – original draft, Writing – review & editing. AG: Supervision, Writing – original draft, Writing – review & editing.
